# Point-of-care ultrasonography in Turkish primary care: a qualitative exploration of practice and experience

**DOI:** 10.1186/s12875-025-03153-w

**Published:** 2025-12-26

**Authors:** Öznur Kübra Odabaş, Duygu Ayhan Başer, Adem Özkara

**Affiliations:** 1 Department of Family Medicine, Ankara Public Health Directorate, Ankara, Türkiye; 2 Department of Family Medicine, Hacettepe University, Ankara, Türkiye; 3 Department of Family Medicine, University of Health Sciences, Bilkent City Hospital, Ankara, Türkiye

**Keywords:** Point-of-Care ultrasonography, Family medicine, Primary care, Diagnostic imaging, Qualitative research

## Abstract

**Background and objective:**

The use of point-of-care ultrasonography (POCUS) by family physicians is growing worldwide, yet remains uncommon in Türkiye. This study aims to explore what motivates family physicians in Türkiye to voluntarily adopt POCUS and how they perceive its benefits and challenges in daily practice.

**Methods:**

This qualitative study used a phenomenological approach and thematic analysis. Ten family physicians from different regions of Türkiye, all with prior POCUS training and at least six months of active use in primary care, were purposively recruited through professional referrals and snowball sampling. Semi-structured interviews were conducted via video conferencing, audio-recorded, and transcribed verbatim. Thematic analysis was performed using MAXQDA 2020. Broad themes guided interview development, while subthemes emerged inductively during coding and were refined through iterative analysis and peer feedback.

**Results:**

Physicians’ motivations to use POCUS were driven by clinical needs, professional curiosity, and skills gained during residency or short courses. Applications included abdominal pain evaluation, pregnancy screening, and chronic disease management. Participants described a wide range of devices used during their learning and daily practice. They viewed POCUS as valuable for improving diagnostic accuracy and patient trust, and several perceived its wider adoption as inevitable in modern primary care. However, they acknowledged increased workload and time pressure in busy primary care settings. Barriers included inconsistent training quality, limited access to equipment, and a lack of institutional support.

**Conclusions:**

POCUS is perceived by Turkish family physicians as a beneficial, patient-centered tool in primary care, particularly in resource-limited settings. Its broader adoption will require structured training, affordable equipment, and supportive health policy to ensure safe, efficient, and sustainable national-level integration.

**Supplementary Information:**

The online version contains supplementary material available at 10.1186/s12875-025-03153-w.

## Introduction

Point-of-care ultrasonography (POCUS) is increasingly used in primary care worldwide as an extension of the physical examination, providing rapid diagnostic insights at the bedside [1–6]. The growth of portable and affordable ultrasound devices has facilitated its integration into general practice in many high-income countries. However, adoption in low- and middle-income countries (LMICs) remains limited, often due to a lack of formal training, regulatory clarity, and access to equipment [7, 8]. In the modern clinical landscape, ultrasonography is no longer confined to radiology departments. A widely accepted term is POCUS, which denotes ultrasound performed by the treating clinician at the point of care, rather than in a remote imaging department [9].

Internationally, ultrasound devices are often grouped into broad tiers, including handheld units, portable/compact systems, and high-end machines, which differ greatly in cost, image quality, and practical usability [10]. In Türkiye, early POCUS adopters frequently rely on whatever device type they can access during residency or obtain personally, making device availability an important contextual factor that shapes both the motivation for, and the feasibility of, POCUS use in primary care.

Family medicine in Türkiye was launched in 2005 under the Health Transformation Program, starting with a pilot and reaching nationwide coverage by 2010 [11]. Since then, efforts have focused on strengthening infrastructure, expanding services, and enhancing physician capacity. Family Health Centers (FHCs), the main entry point to care, often operate with limited diagnostic resources; ultrasonography is typically available only at higher-level facilities.

Following the Health Transformation Program, citizens gained access to primary care services free of charge [12]. Primary care has since been delivered through FHCs and Healthy Life Centers within the scope of public health services, as well as through family medicine outpatient clinics in hospitals staffed exclusively by family medicine specialists. Importantly, the financing and organizational structures of these two models differ. In hospitals, physicians are salaried employees without direct responsibility for service expenses. In contrast, in FHCs, physicians, both specialists and general practitioners are contracted with the Ministry of Health. In FHCs, remuneration includes both salary and operational allowances, making physicians primarily responsible for managing expenses and resources [13]. Understanding this structural distinction is critical, as it shapes how family physicians in different settings may approach the use of ultrasonography. The dual system highlights both opportunities and barriers for integrating POCUS into Turkish primary care, with resource allocation and financial responsibility acting as important contextual factors [14].

International studies suggest that POCUS in primary care can improve diagnostic accuracy, reduce unnecessary referrals, and strengthen patient–physician relationships [3, 15–18]. Some authors have described POCUS as “the new stethoscope”, emphasizing its versatility for applications across nearly all organ systems, from head to toe. These benefits, however, are conditional on factors such as adequate training, device quality, and institutional support [16, 19–22]. In Türkiye, no structured training pathways or formal policies currently exist, creating uncertainty about how family physicians are adopting POCUS in practice and how it fits into the broader health system. This gap provides the rationale for the present study [23].

Despite a growing international body of research on the use of POCUS in primary care, little is known about why some family physicians voluntarily adopt this technology, particularly in health systems such as Türkiye’s, where no formal training pathways, reimbursement structures, or national guidelines currently exist [23–25]. Existing studies primarily focus on diagnostic accuracy, training models, or clinical outcomes; however, the motivational, professional, and practice-related factors influencing early adopters remain underexplored [3, 4, 7, 20]. Notably, no qualitative study to date has examined the perspectives of family physicians using POCUS in Turkish primary care. This gap limits our understanding of what drives clinicians to integrate POCUS into their routine work and how such insights could inform curriculum development, workforce planning, and policy decisions.

This qualitative study focuses on family physicians in Türkiye who have voluntarily integrated POCUS into their daily primary care practice. In this study, POCUS refers to ultrasonographic examinations performed directly by family physicians within their own clinical settings, without referral to secondary or tertiary care. Rather than aiming to capture all possible “experiences,” the study specifically examines the factors that influence family physicians’ decisions to use POCUS in practice and how they perceive its impact on their clinical work. Therefore, this study aims to explore what motivates family physicians in Türkiye to voluntarily adopt POCUS and how they perceive the benefits, challenges, and professional implications of integrating POCUS into primary care.

## Methods

### Study design

This study was guided by a focused aim: to explore what motivates family physicians in Türkiye to voluntarily adopt POCUS and how they perceive its benefits, challenges, and professional implications in primary care.

A qualitative phenomenological approach was used, following the COREQ (Consolidated Criteria for Reporting Qualitative Research) guidelines to ensure methodological rigor [26]. This design was chosen to allow an in-depth understanding of the perspectives and decision-making processes of family physicians who use POCUS in their clinical practice.

### Setting and participants

Ten family physicians, including both specialists and general practitioners, were purposively recruited from different regions of Türkiye through referrals and snowball sampling. Inclusion criteria were: [1] completion of any post-graduate POCUS training [2], at least six months of active clinical use, and [3] active practice in primary care while using POCUS. Although the sample was small, it reflects the limited number of active POCUS users in Türkiye.

Initial contact was made with 13 physicians; three were excluded: two due to time constraints and one because their POCUS practice was limited to a private hospital setting. Similar responses were repeatedly obtained, indicating that data saturation had been reached; therefore, further recruitment attempts were not pursued.

The interview guide was developed based on a review of the existing literature and refined through expert input. It was reviewed and refined but not pilot-tested. It was structured to elicit participants’ motivations for adopting POCUS and their perceptions of its benefits, challenges, and implications for clinical practice.

### Research team and reflexivity

All interviews were conducted by OKO, a female family medicine specialist with personal POCUS experience but with first-time exposure to qualitative research. Except for one participant, the others did not know the researcher prior to the study and had no information about her beyond her professional identity. She received supervision from senior qualitative researchers for interview design, coding, and interpretation. Her background as a POCUS practitioner may have facilitated rapport with participants, while reflexivity was maintained through memo-writing and regular peer debriefing. Throughout the study, the research team reflected on how their clinical backgrounds, prior experiences with POCUS, and expectations could influence data interpretation, and took steps to minimize this influence through continuous reflexive discussions.

### Data collection

Semi-structured interviews were conducted online in Turkish via video conferencing platforms at times convenient to participants, lasting between 30 and 90 min. Before each interview, participants were contacted by phone and informed about the study’s aims and procedures. Written informed consent was obtained (Supplementary File 1). No repeat interviews were conducted. No other individuals were present during the interviews. All sessions were audio-recorded and transcribed verbatim using automated dictation software, followed by manual accuracy checks. Brief field notes were taken during and immediately after each interview to support reflexive interpretation. Participants were invited to review and revise their transcripts. The interview guide is provided in Supplementary File 2.

### Data analysis

Thematic analysis was conducted using MAXQDA 2020 (VERBI Software, Berlin, Germany [27]. The analysis proceeded deductively and inductively, with themes directly reflecting the refined research aim. Coding was inductive, allowing patterns to emerge from the data without a pre-existing framework. All coding, code aggregation, theme development, and text management were performed within MAXQDA. A codebook was maintained and iteratively refined throughout the process (Supplementary File 3). As coding progressed, similar codes were grouped into categories and broader themes; in total, four themes were identified.

A peer researcher independently reviewed the coding and theme development; disagreements were resolved through consensus. Representative quotations are presented in the results and labeled by participant code (e.g., A1, A2). Where minor edits were made to quotations for clarity (e.g., filler words, grammar), the original meaning was preserved.

All analysis was conducted in Turkish. Findings and quotations were translated into English by the corresponding author during manuscript preparation and checked by a bilingual peer researcher. Where direct translation was not possible, conceptual and cultural equivalence was ensured through consensus translation between two bilingual researchers, minimizing the risk of “lost in translation”.

To enhance trustworthiness, preliminary themes were shared with two participants, who confirmed that the interpretations reflected their experiences.

### Ethical considerations

All participants provided written informed consent. Ethical approval was obtained from the University of Health Sciences – Ankara Bilkent City Hospital Ethics Committee (Approval No: TABED1/1022/2025). This study adhered to the principles of the Declaration of Helsinki (World Medical Association).

## Results

A total of ten family physicians participated in the study. Ages ranged from 39 to 60, with POCUS experience ranging from 1 to 30 years. Participant characteristics are summarized in Table 1. Thematic analysis yielded four overarching themes, each with subthemes and illustrative quotations.

**Table 1:** Demographics

Participant number	Age	Gender	Career(Y) (MD/S)	Specialization	Position	POCUS experience(Y)	Daily POCUS usage/ Daily Patient load (average values for the current schedule)***
A1	39	Male	14/10 Y	FM	Assist. Prof.*	10–20	10/100
A2	52	Male	27/24 Y	FM-OB/GYN	Specialist	20–30	60/80
A3	40	Male	16/9 Y	FM	Specialist	5–10	5/100
A4	39	Male	16/3 Y	FM	Specialist	1–5	1/30
A5	36	Female	12/8 Y	FM	Specialist	5–10	1/100
A6	56	Male	24/0 Y	-	MD	5–10	12/90
A7	55	Male	31/25 Y	FM	Specialist	20–30	25/70
A8	60	Male	37/27 Y	FM	Professor**	20–30	1/20
A9	41	Male	16/12 Y	FM	Assist. Prof.*	5–10	12/80
A10	43	Female	19/13 Y	FM	Assist. Prof.*	1–5	2/45

All included participants, including those currently holding academic titles, provide clinical services in primary care settings and gained their practical POCUS experience within these settings

*Y* Years, *MD* Medical Doctor, *S* Specialist, *POCUS* Point of Care Ultrasonography, *FM* Family Medicine, *OB/GYN* Obstetrics and Gynaecologist

* Current position is Associate Professor, and they also serve as mentors in their affiliated clinics. Their primary POCUS experience was gained during earlier periods working in rural settings

** Current position is Professor, and he also serves as a mentor in his affiliated clinic. His primary POCUS experience was gained in his current position

*** Daily patient load reflects the approximate workload during the period in which participants gained majority of their practical POCUS experience

### Theme 1. the process of adopting and internalizing POCUS

Most participants developed interest during residency, particularly during Ob/Gyn and Radiology rotations, often influenced by mentors or unmet clinical needs. One participant recounted that diagnosing gallbladder carcinoma in an early encounter encouraged continued use of ultrasound. Motivations included both personal factors, such as professional growth and satisfaction, and clinical goals such as improving diagnostic accuracy and reducing unnecessary referrals.

Many physicians described POCUS as an extension of the physical examination.

“If I perform an ultrasound when the patient arrives, I feel my physical examination is complete.” (A2).

### Learning pathways and devices used

**Fig. 1:**
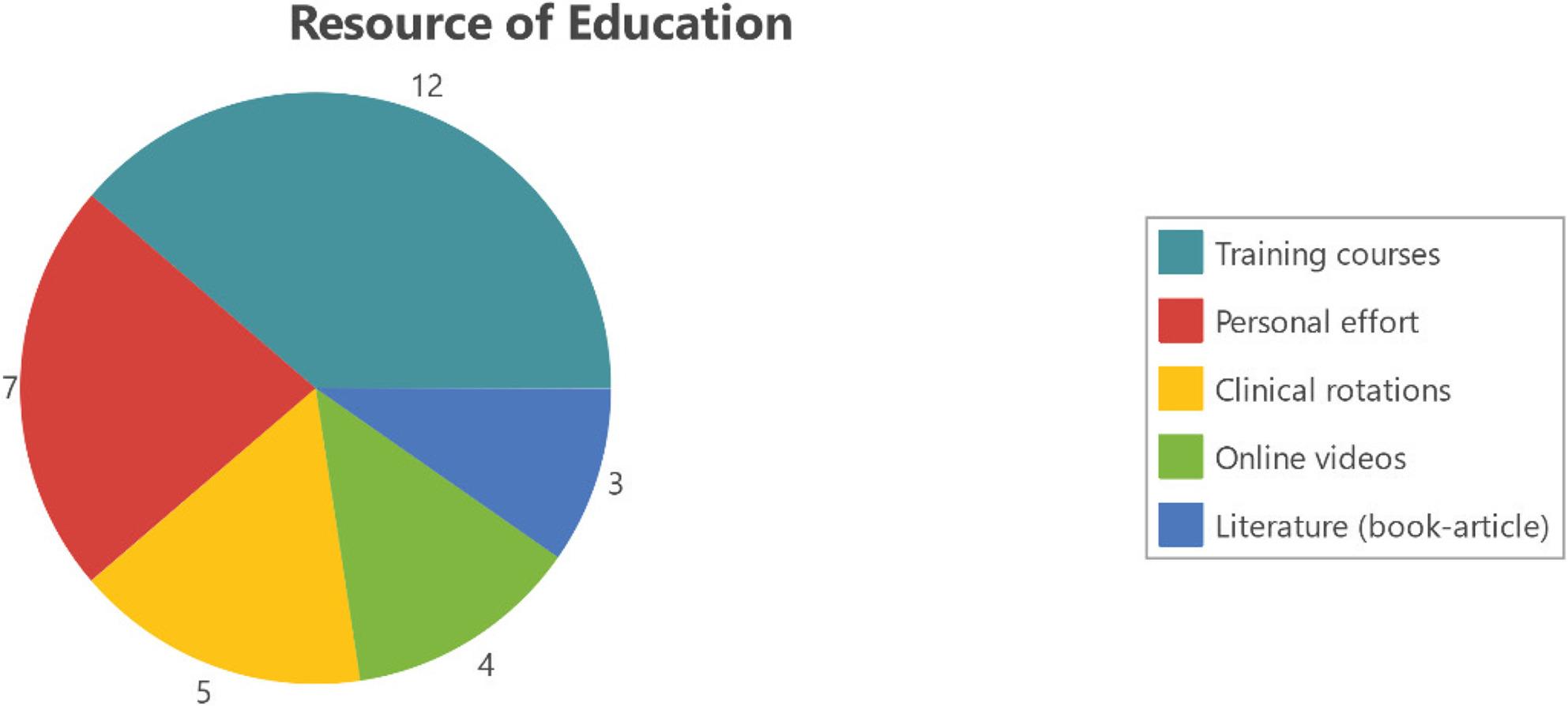
Thematic visualizations of training and usage. Colored segments represent sub-themes within “Training & Usage,” and numbers indicate coding frequency

Training ranged from short courses to online study and peer learning (Fig. 1).

“It is not possible to learn well without firsthand experience. Courses and books are not enough.” (A9).

“I watched over 50 hours of online video just to learn how to hold the probe properly. That was my starting point.” (A5).

**Fig. 2:**
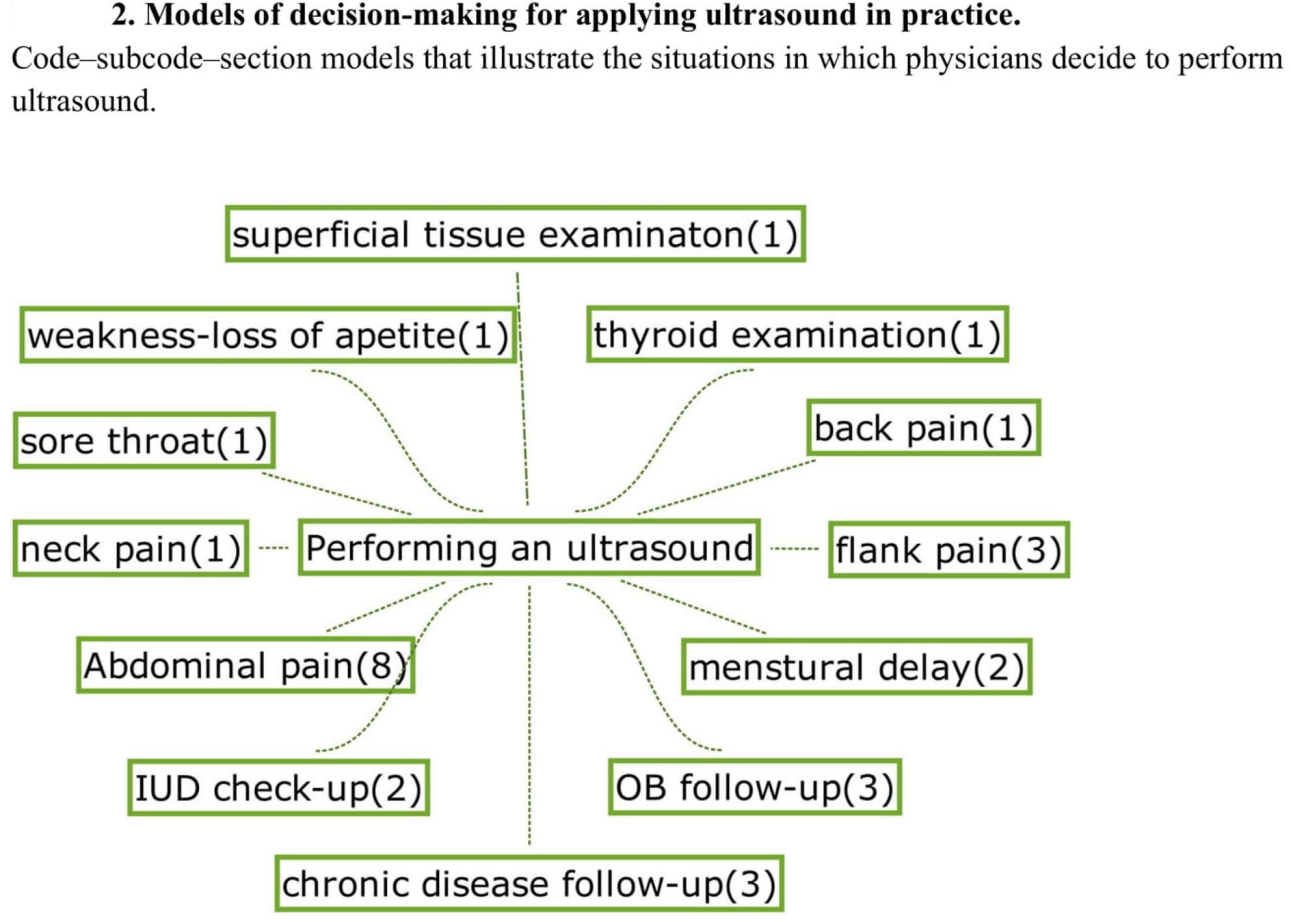
Models of decision-making for applying ultrasound in practice.Code–subcode–section models that illustrate the situations in which physicians decide to perform ultrasound.

Educational content included emergency protocols, echocardiography, and anatomy for nerve blocks. Participants described a wide range of devices used during their learning and daily practice. Many first learned POCUS on various ultrasound machines available in the hospitals where they trained. In their individual practice, most relied on hospital-provided devices, which were often older high-end ultrasound units. A smaller group used personally acquired secondhand high-end machines, while only a few had access to portable cart-based systems or handheld devices. Ultrasound machines were typically obtained either through personal purchase or by borrowing from institutions or colleagues. The most commonly used probe types were convex and linear, reflecting their frequent use in abdominal, lung, thyroid, obstetric, vascular, and urinary scans. Less frequent applications included musculoskeletal, lymph node, cardiac, and ophthalmic scans. Most participants reported a selective, indication-based approach rather than routine scanning (Fig. 2).

Some described personal initiative as key to access:

“We had an ultrasound device that remained unused in the clinic. So I asked, why not me?” (A10).

Participants emphasized that integrating POCUS into workflow improved efficiency and streamlined clinical decision-making.

### Theme 2. professional and clinical implications

POCUS was used for abdominal pain, gynecologic complaints, chronic disease management, lung assessment, and occasional musculoskeletal or vascular evaluations. Workflow integration was flexible. Findings were shared verbally or documented in patient charts.

Participants described the transformative impact of POCUS on clinical practice.

“It was finally seeing what I used to only guess. The image gave me and my patient clarity.” (A8).

A total of 53 pathologies were identified (Supplementary Table 1), including memorable and educational cases such as empyema in a misdiagnosed child and a previously unrecognized 33-week pregnancy (Supplementary Table 2).

Several physicians described emotionally meaningful encounters in which patients expressed gratitude or relief after being examined with ultrasound and noted that it strengthened the therapeutic relationship:

“He says, ‘This doctor cares about me. He did something no one else did.’ His family appreciates it deeply.” (A6).

One system-level advantage frequently noted by participants was the ability to follow a defined patient population over time, which enabled opportunistic screening and early detection. Several physicians explained that having a registered patient list allowed them to apply POCUS more systematically and gradually expand their clinical scope. As one participant described:

“When I first obtained the device, I scanned all my patients. I performed around 1,500 thyroid ultrasounds. Gradually, I started doing abdominal scans as well.” (A3).

Participants also noted that POCUS enhanced their professional identity.

“Do not become a specialist without learning POCUS.” (A7).

Some recalled formative moments with a mix of surprise and excitement:

“I was sitting with assistants and specialists at a congress, and I said that family physicians should also use ultrasound. Everyone laughed. But the very next day, a family physician presented a series of 60 cases he had done with ultrasound.” (A4).

### Theme 3. perceived challenges and barriers

Participants identified multiple challenges that limited the effective use of POCUS in primary care, including high workload, limited clinical space, variability in device quality, anatomic difficulties, and gaps in training. Concerns about diagnostic uncertainty also emerged, particularly among less experienced users:

“Experience is important. Ultrasound is not always precise. Without enough practice or training, we might get the wrong results and cause unnecessary anxiety.” (A8).

Beyond these structural and technical challenges, several physicians described negative emotional experiences that affected their motivation and confidence. The emotional impact of professional criticism was striking for some participants, who recalled moments of disappointment and isolation. One physician noted interpersonal discouragement:

“The most demoralizing comments came from colleagues, saying things like ‘Why are you doing ultrasounds? It is not our job.’” (A9).

**Fig. 3:**
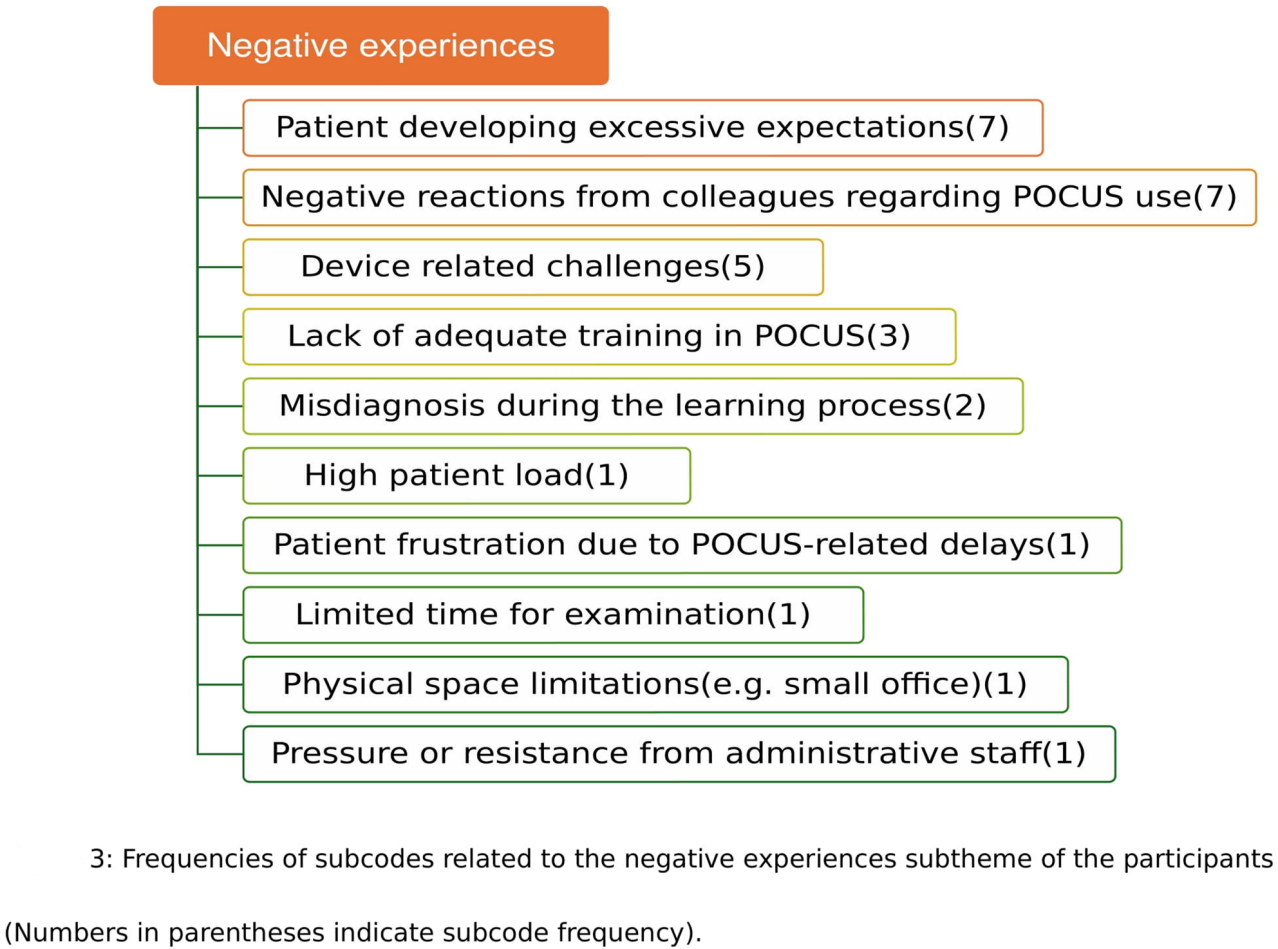
Frequency of subcodes related to the negative experiences subtheme of the participants. The figure summarizes the number of codings assigned to each subcode reported by participants

A visual summary of these negative experiences and their relative frequencies is presented in Fig. 3.

Some physicians also expressed uncertainty about documentation and legal clarity, reflecting concerns about the absence of formal guidance:

“Documentation is tricky. Sometimes I just save the images on my phone for later reflection.” (A6).

### Theme 4. future directions and system-level needs

Participants emphasized the need for structured training, accessible devices, and institutional recognition. Peer support and affordable equipment were seen as essential steps.

Several participants compared the trajectory of family medicine to emergency medicine:

“In 2008, only a few instructors were doing basic ultrasound, but now they offer full courses. Family medicine could follow a similar path.” (A9).

Financial and systemic support were frequently mentioned:

“If the workload in primary care is reduced and physicians receive additional payment when performing ultrasound, it would be easier to get training or purchase devices. Not everyone finds only moral satisfaction enough; some financial incentives are also necessary.” (A8).

Several participants emphasized that the wider adoption of POCUS in primary care requires not only training and equipment but also a shift in system-level thinking about who is allowed to use ultrasound. Some argued that ultrasound should be viewed as a general diagnostic tool rather than a procedure restricted to specific departments.

As one physician expressed, “I see ultrasound as a tool that simplifies diagnosis. Everyone should be able to use it; it does not need to be monopolized by a single specialty.” (A1) This perspective reflects a broader expectation for policies that democratize access to POCUS across the primary care workforce. 

Several participants expressed frustration about the lack of ultrasound exposure during their medical education, describing it as a mismatch between the curriculum and the realities of primary care practice. One physician voiced this sentiment strongly:

“In medical school, even CT and MRI images are explained in detail, but ultrasounds are not shown. It is not hard to provide a basic ultrasound machine to a family doctor in a village, but what is the use of knowing about MRI if we will never have access to one?” (A2).

## Discussion

In recent years, the expansion of POCUS in primary care has accelerated due to rapid technological advances, especially the widespread availability of portable and handheld ultrasound devices [10]. Ultrasound has even been used aboard the International Space Station, demonstrating its feasibility in extreme environments and reinforcing its value as a fundamental diagnostic tool [28, 29]. Despite these global developments, the systematic integration of ultrasound into primary care, especially in Türkiye, remains limited. This gap is not new; although publications on first-contact ultrasound use in general practice date back to the 1970s, the modality has yet to find a stable place in routine primary care, suggesting that long-standing barriers to adoption remain unresolved. Against this backdrop, our findings highlight the need for structural, educational, and policy-level strategies to normalize ultrasound as a routine part of comprehensive primary care.

Consistent with this broader global context, this study provides an in-depth look into how family physicians in Türkiye are integrating POCUS into daily primary care practice, largely in the absence of formal institutional support. The findings align with prior international research suggesting that POCUS can thrive in primary care through self-motivated learning, clinical necessity, and adaptation to local health system constraints [9, 30].

Building on these observations, one of the clearest messages emerging from this study is the transformative effect of POCUS on clinical practice. Participants consistently described ultrasound as “an extension of the physical exam” that not only improved diagnostic precision but also reshaped their professional identity. Similar findings were reported in Denmark and Canada, where POCUS enhanced diagnostic confidence [17, 18].

In parallel with these professional experiences, participants used a wide range of devices from older high-end units to secondhand machines, portable systems, and handheld devices. Such heterogeneity in device availability has also been noted in international primary care studies, reflecting both technological evolution and variations in access across practice setting [2, 31, 32].

Training, however, remained a major gap. While some attended short-term courses, most relied on “learning by doing,” supported by online resources or peer mentoring. This pattern aligns with previous reports describing the limited availability of structured primary care–focused ultrasound curricula and the reliance on self-directed experiential learning among early adopters [20, 22, 23].

Another key issue emerging from our data concerns training pathways and competency development. Participants emphasized that real-life, hands-on practice was essential. Theoretical courses alone were considered insufficient for achieving competence; however, participation in a basic POCUS course was found to significantly increase physicians’ use of POCUS. Longer or repeated sessions may still be necessary to preserve skills and deepen clinical integration [23].

At the same time, professional challenges were also reported. Several participants described discouraging feedback, particularly from colleagues in family medicine. These tensions highlight the absence of clear policy on ultrasound use [33].

Despite these obstacles, participants emphasized diagnostic and relational benefits, from guiding chronic disease management to improving referral quality. POCUS was mostly used for abdominal complaints [34] and also seen as particularly valuable for lung ultrasound in primary care, and potentially for fracture assessment in certain contexts, though rarely applied in practice [35–38].

Similarly, patients’ appreciation was deeply felt. On a broader level, POCUS supported timely access, and gave autonomy to rural physicians [18]. Yet practical limitations, such as limited space and high patient volume, persisted.

From a system-level perspective, in our study, task shifting of POCUS from radiology to primary care clinicians was seen as essential. LMIC studies describe an even broader model, extending to health workers with shorter training and fewer qualifications. Barriers such as limited local leadership, high turnover of trained personnel, training constraints, and lack of algorithms parallel our findings. However, unlike LMIC contexts where unstable electricity and poor internet are major obstacles, Turkish barriers were mainly related to training, device quality, and regulatory clarity [39].

In Türkiye, access to POCUS in primary care is possible either while working in hospital settings or by purchasing a personal device. Some suggested including basic ultrasound devices as standard equipment in FHCs, with subsidies or bulk procurement to reduce costs. Also, family physicians receive no reimbursement for POCUS use in primary care. In contrast, in countries where payment systems exist, billing procedures are often reported to be complex and burdensome, discouraging broader adoption [19].

Regulatory uncertainty also emerged as a major theme. Clear regulatory guidance is essential. Without it, many physicians feel uncertain about the legal scope of ultrasound use in family practice. Overgaard et al. published a comprehensive clinician guide. However, national-level guidelines should go further by addressing not only the scope of practice but also documentation standards, competency assessment, and quality assurance [19, 40].

In response to these challenges, participants proposed practical solutions: affordable portable devices, peer support, and incorporation of ultrasound into medical education. Some compared family medicine to emergency medicine, which developed its own ultrasound pathways over the last 15 years [24, 25, 41]. At the educational level, participants widely recommended integrating POCUS into undergraduate and residency curricula.

Together, these findings underscore the need for structural support to ensure safe, sustainable, and effective POCUS integration in primary care.

In conclusion, Turkish family physicians are working actively to improve patient care by integrating ultrasound into their clinical routines, despite limited resources. Their experiences highlight why ultrasound should be accepted and supported in primary care, not only as a diagnostic tool but also as a means of strengthening professional identity, improving system efficiency, and building trust with patients. Participants believed that personal motivation alone would not be enough to make POCUS a routine part of family medicine. Support from the Ministry of Health, combined with structured training, accessible equipment, and a clear regulatory framework, would ensure that POCUS use is both safe and sustainable, while maximizing its benefits for patients and the healthcare system.

### Strengths and limitations

This study is limited by its sample size of ten physicians, which, although sufficient for thematic saturation in qualitative research, may not capture the full diversity of experiences across Türkiye. However, this number also reflects the limited pool of family physicians in the country who actively use POCUS in primary care. Recruiting participants was challenging, as the study design required physicians to dedicate time for long and in-depth interviews, which was difficult to accommodate in their busy clinical schedules. Two eligible physicians could not participate due to time constraints, yet the ten who volunteered showed remarkable dedication and generosity in contributing their time. Simply documenting the presence of at least ten such physicians is noteworthy, as it demonstrates that POCUS is already being practiced in Turkish primary care and highlights its potential value for colleagues, policymakers, and educators.

Another limitation is that most participating physicians had long professional experience and had worked in both rural and urban settings during their careers, making it difficult to clearly separate experiences by context. To minimize this ambiguity, participants were reminded to focus on the period in which they used ultrasonography most intensively. Further, all participants were active users of ultrasonography, which may introduce selection bias toward more favorable perceptions. Data were also self-reported and thus subject to recall or social desirability bias.

At the same time, the study has several strengths. It is, to our knowledge, the first qualitative exploration of POCUS use in Turkish primary care and provides insights that have not been captured in previous quantitative reports. The inclusion of physicians with substantial professional experience, often across both rural and urban contexts, allowed for a broad perspective on structural facilitators and barriers. Importantly, all physicians reported that their core ultrasound experience was acquired in rural areas, often during compulsory service. This characteristic may complement findings from comparable studies, such as the Danish qualitative study, by highlighting how structural and contextual barriers shape ultrasound use in primary care [17]. Moreover, the study adhered to established qualitative standards, using COREQ criteria and a peer-reviewed coding process to enhance credibility. Finally, particular attention was paid to situating findings within a body of up-to-date international literature, ensuring that interpretations and comparisons reflect the current state of knowledge in this rapidly evolving field.

## Supplementary Information

Supplementary Material 1.

Supplementary Material 2.

Supplementary Material 3.

Supplementary Material 4.

Supplementary Material 5.

Supplementary Material 6.

## Data Availability

The datasets generated and/or analyzed during the current study are not publicly available due to confidentiality of interview transcripts but are available from the corresponding author on reasonable request.
