# Supplementary material for: Point-of-care ultrasonography in Turkish primary care: a qualitative exploration of practice and experience
Source: BMC Prim Care. 2025 Dec 26;27:26. doi: 10.1186/s12875-025-03153-w (PMC12849191; doi:10.1186/s12875-025-03153-w)
Supplement: Supplementary file 1 — Supplementary Material 1. [file 12875_2025_3153_MOESM1_ESM.docx]

# INFORMED CONSENT FORM

**Point-of-Care Ultrasonography in Turkish Primary Care: A Qualitative Exploration of Practice and Experience**

## (Researcher’s Explanation)

Dear Participant,

We are inviting you to participate in a study that aims to explore the experiences of family physicians regarding the use of POCUS. Participation in this study is entirely voluntary. Before you decide, it is important that you understand the purpose and the procedures of the study so you can make an informed decision. Please read the information below carefully and do not hesitate to ask questions.

## Purpose of the Study

The aim of this research is to scientifically document and share the experiences of primary care physicians who actively use POCUS in their clinical practice.

## Procedures

If you agree to participate, the responsible researcher will conduct an interview using a 15-question data collection form. The interview will be conducted face-to-face or via video conferencing, and your responses will be recorded electronically and transcribed. Notes on nonverbal expressions may also be taken. The interview will take approximately 40–60 minutes. There is no compensation or cost associated with participation.

Audio recordings will be securely stored and destroyed after transcription. If you do not consent to audio recording, written notes will be taken instead.

## Confidentiality and Use of Data

All personal data will be kept confidential. Your identity will not be disclosed in any report or publication. Data may be reviewed by ethics committees or regulatory authorities if required. You are free to withdraw from the study at any time. However, once your data has been anonymized and included in the analysis, it cannot be removed.

## Voluntary Participation

Participation is entirely voluntary. Declining to participate will not result in any negative consequences. You may also withdraw your consent at any point during the study.

## Participant Declaration

I have been informed about the study titled ‘**Point-of-Care Ultrasonography in Turkish Primary Care: A Qualitative Exploration of Practice and Experience’**

by Family Medicine Specialist Dr. Öznur Kübra Odabaş. I understand that:

- My participation is voluntary and I may withdraw at any time.

- The interview may be audio recorded with my permission.

- The audio recordings will be transcribed and then destroyed securely.

- My identity will remain confidential in all publications and data sharing.

- The results will be used for educational and scientific purposes only.

If I have questions, I can contact the researcher at +90 543 151 51 03.

By participating in the interview, I confirm that I have read and understood this form, and I give consent for the analysis and publication of the information I provide.
Participant Name & Surname:

Date:

Researcher Name & Surname: Dr. Öznur Kübra Odabaş

Date:
