# Supplementary material for: Point-of-care ultrasonography in Turkish primary care: a qualitative exploration of practice and experience
Source: BMC Prim Care. 2025 Dec 26;27:26. doi: 10.1186/s12875-025-03153-w (PMC12849191; doi:10.1186/s12875-025-03153-w)
Supplement: Supplementary file 2 — Supplementary Material 2. [file 12875_2025_3153_MOESM2_ESM.docx]

**Point-of-Care Ultrasonography in Turkish Primary Care: A Qualitative Exploration of Practice and Experience**

**RESEARCH DATA COLLECTION FORM**

GENERAL INFORMATION

1. What is your date of birth and graduation year? Gender: M/F

2. What is your current field of practice, geographic region, and patient population?

MOTIVATION

3. How did your interest in point of care ultrasonography (POCUS) begin?

4. How did you decide to incorporate POCUS into your clinical practice?

5. What type of POCUS device did you initially use?

EDUCATION

6. Where and for how long did you receive POCUS training?

7. Could you detail the content of your training?

8. How long have you been performing POCUS?

9. Are you affiliated with any group, association, or working team related to POCUS?

POCUS USE

10. What are the features of the POCUS device(s) you use?

11. How often do you use POCUS in your daily practice?

12. Which anatomical regions do you scan most frequently?

13. Are there any regions you avoid scanning? Why?

14. Are there any patient groups you avoid scanning? Why?

CLINICAL EXPERIENCE

15. Could you share a patient case where you decided to use POCUS in your daily practice?

16. Do you primarily use POCUS for diagnostic indications or screening?

17. Do you ever expand your scan beyond the initially planned region?

18. Are you able to complete the POCUS scan within your planned time frame?

19. Does performing POCUS affect the scheduled appointment times of your patients?

20. Does performing POCUS affect your patient workload?

21. Do you document your findings?

22. When referring a patient to secondary care, do you send a note or report including POCUS findings to the specialist?

DIAGNOSTIC PROCESS

23. To what extent do you think your POCUS findings contribute to your diagnoses?

24. Can you give an example of a case where POCUS helped in your diagnostic process?

25. Can you give an example where POCUS did not contribute to your diagnostic process?

26. What pathologies have you diagnosed using POCUS?

27. Do you have any noteworthy or educational experiences related to POCUS -based diagnosis?

28. Can you share a positive case example where POCUS contributed to your clinical experience?

29. Can you share a negative case example where POCUS affected your clinical experience?

COMMUNICATION

30. How do you inform patients before performing POCUS?

31. Do you obtain verbal or written consent from patients before performing POCUS?

32. Have you ever had a patient refuse POCUS? Why?

33. Have you experienced patients having high or unrealistic expectations regarding POCUS?

COMPETENCE

34. Do you follow any protocol while performing POCUS?

35. Do you feel confident in your POCUS skills?

36. What do you do when you feel uncertain during an examination?

OUTCOMES

37. How does your POCUS use affect the opinion of specialist physicians?

38. How does your POCUS use affect the doctor-patient relationship?

39. Have you ever felt that POCUS diverted your attention from the main complaint?

EXPERIENCE

40. What challenges have you encountered during your time using POCUS?

41. Do you have any knowledge or experience about the use of POCUS in primary care in countries outside of Turkey?

42. Do you have any publications, research, or case reports related to POCUS?

PREDICTIONS

43. What do you think are the potential benefits of widespread POCUS use by family physicians?

44. What do you think are the potential harms?

45. What do you think are the possible challenges?

46. What kinds of reactions do you expect from other specialties?

47. What are your predictions about the future of POCUS use in primary care in Turkey?

SUGGESTIONS & GOALS

48. What are your suggestions for physicians interested in POCUS?

49. What are your personal goals regarding POCUS?
