# Supplementary material for: Point-of-care ultrasonography in Turkish primary care: a qualitative exploration of practice and experience
Source: BMC Prim Care. 2025 Dec 26;27:26. doi: 10.1186/s12875-025-03153-w (PMC12849191; doi:10.1186/s12875-025-03153-w)
Supplement: Supplementary file 3 — Supplementary Material 3. [file 12875_2025_3153_MOESM3_ESM.docx]

|  | **Coded Sections** |
| --- | --- |
| Hepatobiliary system pathologies   - Gallbladder pathologies (mass, stones)-9 - Hepatosteatosis-1 - Liver hemangioma-1 - Pancreatic nodule-1 | 12 |
| Genitourinary system pathologies   - Urinary system (stone, mass, hydronephrosis, dilatation)-7 - Benign prostatic hyperplasia-2 - Bladder tumor-1 - Renal mass-1 - Testicular tumor-1 | 12 |
| Obs/Gyn pathologies   - Amniotic fluid insufficiency-2 - Myoma-2 - Ovarian cancer, cyst-2 - Pcos-1 - Ectopic pregnancy rupture-1 - Prolapse in pregnancy-1 | 9 |
| Thyroid pathologies(Thyroid hyperplasia- Planjon goiter) | 6 |
| Superficial tissue pathologies   - Dermoid cyst-lipoma-2 - Lymphadenopathy-2 | 4 |
| Vascular pathologies   - Aortic aneurysm-2 - Carotid plaque-1 - Splenic artery aneurysm-1 | 4 |
| Lung pathologies   - Pleural Empyema-1 - Lung Malignancies-1 | 2 |
| GIS pathologies   - Stomach carcinoma-1 - Pediatric intussusception-1 | 2 |
| Cardiac pathology (cardiac tamponade) | 1 |
| Joint pathology (developmental hip dislocation) | 1 |
| TOTAL | 53 |

**Supplementary Table 1. Frequencies of codes in the detected pathologies sub-theme based on section-level coding**
