# Supplementary material for: Point-of-care ultrasonography in Turkish primary care: a qualitative exploration of practice and experience
Source: BMC Prim Care. 2025 Dec 26;27:26. doi: 10.1186/s12875-025-03153-w (PMC12849191; doi:10.1186/s12875-025-03153-w)
Supplement: Supplementary file 4 — Supplementary Material 4. [file 12875_2025_3153_MOESM4_ESM.docx]

# Codebook

This codebook presents the hierarchical structure of themes, subthemes, and codes derived from the thematic analysis of interviews conducted with family physicians who use ultrasonography in primary care settings in Türkiye. Each entry is provided in English to facilitate dissemination and transparency. A total of 712 codes were used.

**Theme 1: The process of adopting and internalizing POCUS**

## 1.1 Interest and Motivation

### 1.1.1 Origins of Interest in POCUS

- During medical school (includes emergency and OB-GYN rotations)
- After graduation
- During residency (positive reinforcement from seniors)
- USG exposure in residency rotations (Obstetrics, Endocrinology, Radiology)
- General interest in medical devices

### 1.1.2. Decision to Use POCUS

- Advice from colleagues
- Device awareness
- Clinical need and context
- Observing peers using POCUS
- Recognizing clinical benefits

### 1.1.3. Purpose of Using POCUS

- Social goals (health system contribution, patient benefit)
- Personal goals (efficiency, job satisfaction, professional development, curiosity, experience)

## 1.2. Training

### 1.2.1. Training Sources

- Online videos
- Literature (books, articles)
- Courses (TATD, TMFTV, congresses)
- Clinical rotations
- Self-directed learning

### 1.2.2. Training Content

- POCUS protocols
- Echocardiography
- Anatomy and nerve blocks
- Emergency courses

## 1.3. POCUS Usage

### 1.3.1. Technical Aspects

- Portable devices
- High-end devices
- Variable devices
- Hands-on devices

### 1.3.2. Access to Devices

- Through colleagues
- Buying second-hand

### 1.3.3. Body Areas of Application

- Rarely scanned regions (lymph nodes, eye, etc.)
- Common regions (abdomen, thyroid, lungs, OB-GYN, etc.)

## 1.4. Clinical Workflow

### 1.4.1. Patient Scenarios

- Pregnancy suspicion, thyroid problems, abdominal pain, etc.

### 1.4.2. Documentation

- Saving USG images
- Brief EHR notes
- Verbal communication with patients

### 1.4.3. POCUS Plan Compliance

- Patient waiting time
- Scope and duration issues
- Referrals and follow-up
- Informing referral centers

### 1.4.4. POCUS Application Preferences

- Indication-based
- Screening
- Broad use
- Selective use
- As part of physical exam

## 1.5. Diagnostic Process

### 1.5.1. Contribution to Diagnosis

- Major contribution
- Limited contribution

### 1.5.2. Detected Pathologies

- Cardiac, hepatobiliary, thyroid, vascular, etc.

### 1.5.3. Educational Cases

## 1.6. Communication

**1.6.1. Informing Patients**

**1.6.2. Patient Consent**

## 1.7. Competency

### 1.7.1. Perceived Competency

- Feeling competent
- Feeling incompetent

### 1.7.2. Protocol Use

- Following course-based protocols
- Online protocols
- No specific protocol

**Theme 2: Professional and clinical implications**

### 2.1. Positive Experiences

- Confirmed findings
- Identifying diagnoses that were previously overlooked in the clinical encounter
- Appreciation from specialists
- Referrals from other departments

### 2.2. Systemic Outcomes

- Cost-effectiveness
- Decreased unnecessary medication
- Qualified referrals
- Receiving recognition from upper-level authorities.

### 2.3. Patient-Related Outcomes

- Earlier diagnosis
- Trust and satisfaction
- Patients feeling they receive special attention.
- Patients showing respectful behavior toward the physician.
- An increase in patients’ trust toward the physician
- Patients becoming overly demanding.

### 2.4. Physician-Related Outcomes

- Facilitating diagnosis, treatment, and follow-up for the patient
- Increased physician self-confidence
- Projecting an image of being up-to-date and continuously improving as a physician
- A positive contribution to the patient–physician relationship
- Professional growth
- Burnout prevention
- Increased workload
- Losing focus from primary complaint

### 2.5. Advantages in Primary Care Setting

- Being familiar with the patient’s full medical background
- Immediate in-room access to ultrasonography
- The opportunity to screen and closely follow a defined population

### Theme 3: Perceived challenges and barriers

### 3.1. Negative Experiences

- Patients developing excessive expectations
- Negative reactions from colleagues regarding POCUS use
- Device-related challenges
- Lack of adequate training in POCUS
- Misdiagnosis during the learning process
- High patient load
- Patient frustration from POCUS-related delays
- Limited time for examination
- Physical space limitations (e.g.small office)
- Pressure or resistance from administrative staff

### 3.2. Reactions from Other Specialties

- Initial skepticism
- Reduced specialist workload

### 3.3. Risks and Potential Challenges

- Institutional resistance
- Cost and training issues
- Risks related to POCUS practice itself

### 3.4. Challenges in Primary Care Setting

- Lack of adequate physical resources, including limited space and equipment
- High patient workload

**Theme 4: Future directions and system-level needs for integrating POCUS**

### 4.1. Future of Family Medicine

- Policy-dependent adoption
- Positive/negative expectations

## 4.2. Recommendations

### Device Acquisition

### Self-Training Methods

### Motivation and Initiative

### Practical Warnings

### Defining Professional Boundaries

## 4.3. Criticisms

- Lack of training
- Lack of institutional support
- Financial issues
- Resistance from academia and associations

## 4.4 Personal Goals

- Becoming professional in POCUS
- Teaching residents
- Acquiring portable or advanced devices
- Continuing education
