# Supplementary material for: Point-of-care ultrasonography in Turkish primary care: a qualitative exploration of practice and experience
Source: BMC Prim Care. 2025 Dec 26;27:26. doi: 10.1186/s12875-025-03153-w (PMC12849191; doi:10.1186/s12875-025-03153-w)
Supplement: Supplementary file 5 — Supplementary Material 5. [file 12875_2025_3153_MOESM5_ESM.docx]

# COREQ 32-Item Checklist (Tong et al., 2007)

This checklist was completed using the Consolidated Criteria for Reporting Qualitative Research (COREQ): a 32-item checklist for interviews and focus groups, as recommended by the EQUATOR Network. The corresponding page numbers in the manuscript where each item is addressed are indicated below.

Checklist completed by: Oznur Kubra Odabas

Date: 1 December 2025

Journal: BMC Primary Care

Manuscript title: Point-of-Care Ultrasonography in Primary Care: A Qualitative Exploration of Practice and Experience

| No. | Item | Guide questions / description | Page number |
| --- | --- | --- | --- |
| 1 | Interviewer/ facilitator | Which author/s conducted the interview or focus group?/Oznur Kubra Odabas | Page: 4  Methods/ Research Team and Reflexivity Section |
| 2 | Credentials | What were the researcher's credentials? e.g. PhD, MD | Author roles and affiliations are listed on the Title Page. |
| 3 | Occupation | What was their occupation at the time of the study? | Author roles and affiliations are listed on the Title Page. |
| 4 | Gender | Was the researcher male or female?/Female | Page: 4  Methods/ Research Team and Reflexivity Section |
| 5 | Experience and training | What experience or training did the researcher have? The interviewer was a family medicine specialist with personal POCUS experience and received supervision from senior qualitative researchers, as this was her first qualitative study. | Page: 4  Methods/ Research Team and Reflexivity Section |
| 6 | Relationship established | Was a relationship established prior to study commencement?/ Except for one participant, the others did not know the researcher prior to the study | Page: 4  Methods/ Research Team and Reflexivity Section |
| 7 | Participant knowledge of the interviewer | What did the participants know about the researcher? e.g. personal goals, reasons for doing the research/ Participants had no information about her beyond her professional identity. | Page: 4  Methods/ Research Team and Reflexivity Section |
| 8 | Interviewer characteristics | What characteristics were reported about the interviewer/facilitator? e.g. bias, assumptions, reasons and interests in the research topic/ The interviewer was a family physician with POCUS experience. Potential biases were mitigated through reflexive notes and peer debriefing. | Page: 4  Methods/ Research Team and Reflexivity Section |
| 9 | Methodological orientation and theory | What methodological orientation was stated to underpin the study? e.g. grounded theory, discourse analysis, ethnography, phenomenology, content analysis/ A qualitative phenomenological approach was used | Page: 4  Methods/Study Design Section |
| 10 | Participant selection | How were participants selected? e.g. purposive, convenience, consecutive, snowball/ Physicians, were purposively recruited through referrals and snowball sampling | Page: 4  Methods/ Setting and Participants Section |
| 11 | Method of approach | How were participants approached? e.g. face-to-face, telephone, mail, email/ Interviews were conducted online | Page: 4  Methods/ Data Collection Section |
| 12 | Sample size | How many participants were in the study?/ Ten family physicians were in the study | Page: 4  Methods/ Setting and Participants Section |
| 13 | Non-participation | How many people refused to participate or dropped out? Reasons?/ Three of them dropped out: two due to time constraints and one because their POCUS practice was limited to a private hospital setting | Page: 4  Methods/ Setting and Participants Section |
| 14 | Setting of data collection | Where was the data collected? e.g. home, clinic, workplace/ Interviews were conducted online | Page: 4  Methods/ Data Collection Section |
| 15 | Presence of non-participants | Was anyone else present besides the participants and researchers?/ No. Only the interviewer and participant were present during the interviews. | Page: 5  Methods/ Setting and Participants Section |
| 16 | Description of sample | What are the important characteristics of the sample? e.g. demographic data, date/ Participants were family physicians from different regions of Türkiye, aged 39–60, with 1–30 years of POCUS experience. All had completed some form of postgraduate POCUS training and had at least six months of active use in primary care. | Page: 4  Methods/ Setting and Participants Section |
| 17 | Interview guide | Were questions, prompts, guides provided by the authors? Was it pilot tested? It was reviewed and refined but not pilot-tested. | Interview guide is available as a Supplementary File 2. |
| 18 | Repeat interviews | Were repeat interviews carried out? If yes, how many?/ No repeat interviews were conducted. | Page: 5  Methods/ Data Collection Section |
| 19 | Audio/visual recording | Did the research use audio or visual recording to collect the data?/ All sessions were audio-recorded | Page: 5  Methods/ Data Collection Section |
| 20 | Field notes | Were field notes made during and/or after the interview or focus group?/ es. Brief field notes were made during and immediately after each interview. | Page: 5  Methods/ Data Collection Section |
| 21 | Duration | What was the duration of the interviews or focus group?/ Interviews lasted between 30 and 90 minutes. | Page: 4  Methods/ Data Collection Section |
| 22 | Data saturation | Was data saturation discussed?/ Similar responses were repeatedly obtained, indicating that data saturation had been reached. | Page: 4  Methods/ Setting and Participants Section |
| 23 | Transcripts returned | Were transcripts returned to participants for comment and/or correction?/ Participants were invited to review and revise their transcripts. | Page: 5  Methods/ Data Collection Section |
| 24 | Number of data coders | How many data coders coded the data?/ Coding was conducted by the first researcher and independently reviewed by a peer researcher. | Page 5  Methods/ Data Analysis Section |
| 25 | Description of the coding tree | Did authors provide a description of the coding tree?/ yes. | Codebook is included as a Supplementary File 3 |
| 26 | Derivation of themes | Were themes identified in advance or derived from the data?/ Themes were primarily derived inductively from the data; no predefined thematic structure was imposed. Broad areas of inquiry guided the interviews, but final themes emerged through iterative coding and analysis. | Page 5  Methods/ Data Analysis Section |
| 27 | Software | What software, if applicable, was used to manage the data?/ MAXQDA 2020 | Page 5  Methods/ Data Analysis Section |
| 28 | Participant checking | Did participants provide feedback on the findings?/ Participants were invited to review and revise their transcripts. | 5  Methods/ Data Collection |
| 29 | Quotations presented | Were participant quotations presented to illustrate the themes/findings? Was each quotation identified? e.g. participant number/ Yes — participant quotations were presented throughout the Results section to illustrate key themes, and each quotation was clearly identified using participant codes (e.g., A1, A2). | Page 5  Methods/ Data Analysis Section |
| 30 | Data and findings consistent | Was there consistency between the data presented and the findings?/ Yes. The findings were directly supported by participant quotations, and all interpretations were grounded in the data. | Page 5-8  Results Section |
| 31 | Clarity of major themes | Were major themes clearly presented in the findings? Yes | Page 5-8  Results Section |
| 32 | Clarity of minor themes | Is there a description of diverse cases or discussion of minor themes?/ Yes. Minor themes and diverse cases were included, such as less common clinical applications (e.g., musculoskeletal or ophthalmic scans) and rare but illustrative cases (e.g., misdiagnosed empyema, unrecognized pregnancy). These appear in the Results section. | Page 5-8  Results Section |
