# Supplementary material for: Point-of-care ultrasonography in Turkish primary care: a qualitative exploration of practice and experience
Source: BMC Prim Care. 2025 Dec 26;27:26. doi: 10.1186/s12875-025-03153-w (PMC12849191; doi:10.1186/s12875-025-03153-w)
Supplement: Supplementary file 6 — Supplementary Material 6. [file 12875_2025_3153_MOESM6_ESM.docx]

| **Patient's complaint-Final diagnosis** |
| --- |
| Abdominal pain - seminoma metastasis  "...I evaluated a patient who presented with abdominal pain. Normally, diagnosing appendicitis is very, very simple for me. In my 20 years, I've probably diagnosed appendicitis more times than I can count. A young patient came to me and I detected this irregular, circumscribed, and internally heterogeneous, complex mass in the right lower quadrant. The general surgeon had great confidence in me. I'd never disappointed him before, but then the surgeon called and said the patient had a tumor. The patient doesn't have a right testicle. It turns out he had surgery for seminoma, and the image I saw was metastasis. This patient had surgery for seminoma, but he hadn't told me. (A2)" |
| Detection of heartbeats that cannot be detected by fetal doppler  "I have also detected a heartbeat with a probe when a patient came to me complaining that the baby wasn't moving, even though no heartbeat was detected with a fetal Doppler. (A4)" |
| Inpatient who received treatment with the diagnosis of pneumonia but did not recover – empyema  "...A 7-8-year-old child was admitted to the pediatric ward with pneumonia during my residency. I suggested to the doctor that we perform an ultrasound on this child. The doctor said there was air in the lungs, but an ultrasound wouldn't show air. So, what he meant was, there's no such thing as a lung ultrasound... The patient's fever didn't drop, so the doctor gave me permission to take him for an ultrasound. It wasn't pneumonia, it was empyema. We called the thoracic surgeon, who inserted a tube, and two days later, the child's fever dropped. There are no criteria to distinguish pneumonia from empyema, or rather, effusion, on a chest X-ray. Testing for this with an ultrasound takes 10 seconds. (A7)" |
| Difficulty in swallowing - planjon goiter |
| Buzzing in the ear - myoma |
| Delayed menstrual period - 33 weeks pregnant  "A patient came in and had a pregnancy test in rural settings. She was pregnant, but she didn't know the week of pregnancy. I said, 'Let's check with an ultrasound, and it showed 33 weeks.'" (A10) |

**Supplementary Table 2. Educational Case Examples Shared by Participants.**

(Code-based table of the educational case sub-theme; representation related to the reason for application and final diagnosis)
